# Correction: Aging and Hypertension among the Global Poor: Panel Data Evidence from Malawi

**DOI:** 10.1371/journal.pgph.0001811

**Published:** 2023-04-04

**Authors:** The PLOS Global Public Health Staff

## Notice of republication

This article was republished on December 19, 2022 to replace the incorrect editor (“PLOS Editor Invitation”) with the correct editor (Collins Otieno Asweto). The publisher apologizes for the errors. Please download this article again to view the correct version. The originally published, uncorrected article and the republished, corrected articles are provided here for reference.

## Supporting information

S1 File Originally published, uncorrected article. (PDF)

S2 File Republished, corrected article. (PDF)
