# Supplementary material for: Correction: Aging and Hypertension among the Global Poor: Panel Data Evidence from Malawi
Source: PLOS Glob Public Health. 2023 Apr 4;3(4):e0001811. doi: 10.1371/journal.pgph.0001811 (PMC10072472; doi:10.1371/journal.pgph.0001811)
Supplement: S1 File — (PDF) [file pgph.0001811.s001.pdf]

PLOS GLOBAL PUBLIC HEALTH

RESEARCH ARTICLE

Aging and hypertension among the global poor—Panel data evidence from Malawi

Iliana V. Kohler[1], Nikkil Sudharsanan[2], Chiwoza Bandawe[3], Hans-Peter Kohler[4]*

1 Population Studies Center, University of Pennsylvania, Philadelphia, PA, United States of America, **2** Department of Sport and Health Sciences, Technical University of Munich, Munich, Germany, **3** Department of Mental Health, Kamuzu University of Health Sciences, Blantyre, Malawi, **4** Population Aging Research Center and Department of Sociology, University of Pennsylvania, Philadelphia, PA, United States of America

* hpkohler@pop.upenn.edu

Abstract

Hypertension is a rapidly growing disease burden among older persons in low-income countries (LICs) that is often inadequately diagnosed and treated. Yet, most LIC research on hypertension is based on cross-sectional data that does not allow inferences about the onset or persistence of hypertension, its correlates, and changes in hypertension as individuals become older. The Mature Adults Cohort of the Malawi Longitudinal Study of Families and Health (MLSFH-MAC) is used to provide among the first panel analyses of hypertension for older individuals in a sub-Saharan LIC using blood pressure measurements obtained in 2013 and 2017. We find that high blood pressure is very common among mature adults aged 45+, and hypertension is more prevalent among older as compared to middle-aged respondents. Yet, in panel analyses for 2013–17, we find no increase in the prevalence of hypertension as individuals become older. Hypertension often persists over time, and the onset of hypertension is predicted by factors such as being overweight/obese, or being in poor physical health. Otherwise, however, hypertension has few socioeconomic predictors. There is also no gender differences in the level, onset or persistence in hypertension. While hypertension is associated with several negative health or socioeconomic consequences in longitudinal analyses, cascade-of-care analyses document significant gaps in the diagnosis and treatment of hypertension. Overall, our findings indicate that hypertension and related high cardiovascular risks are widespread, persistent, and often not diagnosed or treated in this rural sub-Saharan population of older individuals. Prevalence, onset and persistence of hypertension are common across all subgroups—including, importantly, both women and men. While age is an important predictor of hypertension risk, even in middle ages 45–55 years, hypertension is already widespread. Hypertension among adults aged 45+ in Malawi is thus more similar to a "generalized epidemic" than in high-income countries where cardiovascular risk has strong socioeconomic gradients.

Introduction

Hypertension is not only widespread in higher-income countries. To the contrary, levels of hypertension are actually often higher in sub-Saharan African (SSA) and low- and middle-

OPEN ACCESS

Citation: Kohler IV, Sudharsanan N, Bandawe C, Kohler H-P (2022) Aging and hypertension among the global poor—Panel data evidence from Malawi. PLOS Glob Public Health 2(6): e0000600. https://doi.org/10.1371/journal.pgph.0000600

Editor: PLOS Editor Invitation, Straive, PHILIPPINES

Received: February 10, 2022

Accepted: May 19, 2022

Published: June 28, 2022

Copyright: © 2022 Kohler et al. This is an open access article distributed under the terms of the Creative Commons Attribution License, which permits unrestricted use, distribution, and reproduction in any medium, provided the original author and source are credited.

Data Availability Statement: Data has been uploaded or can be accessed on OSFHome via direct link: https://osf.io/yx4d5/.

Funding: This work was supported by the National Institute of Child Health and Human Development (NICHD, Grant# R01 HD053781, R01 HD087391, all to HPK); the National Institute on Aging (NIA, Grant# P30 AG12836 and R21 AG053763, all to HPK); the NICHD Population Research Infrastructure Program (Grant# R24 HD-044964 to HPK); and the Swiss Programme for Research on Global Issues for Development (SNF r4d Grant

400640-160374 to IVK and HPK). The funders had no role in study design, data collection and analysis, decision to publish, or preparation of the manuscript.

Competing interests: The authors have declared that no competing interests exist.

income countries (LMICs) as compared to high-income countries (HICs) (Fig 1A). Among adults over the age of 18 in SSA as a whole, despite an overall young age structure, more than 20% of men and women have hypertension (systolic BP $\geq$ 140 mmHg or diastolic BP $\geq$ 90 mmHg) [1, 2]. However, the diagnosis and treatment of this widespread hypertension is often inadequate [3]. Moreover, the high prevalence of hypertension in SSA and/or LMIC contrasts with an often low prevalence of "classic" risk factors (i.e., high rates of obesity, unhealthy life styles and reduced physical activity) [1, 2, 4], indicating that hypertension in LIC has partially different causes than hypertension in high-income contexts. In global comparison, for example, women in SSA have among the highest prevalence of hypertension, while they also are among the leanest in the world (Fig 1A+1B).

This prevalence and number of individuals in need of hypertension care is expected to grow substantially in LMICs [2, 5–7]. This rise in hypertension is related to a broader shift in the burden of disease in LMICs towards non-communicable diseases (NCDs) that have become leading causes of adult morbidity and mortality in sub-Saharan African (SSA) countries [8]. The shifting disease burden towards NCDs, along with a gradual aging of global populations [9], has been described as a *frontier* in global health research [10] that is likely to regain focus once the Covid-19 pandemic has receded [7, 11].

Despite the importance of understanding and addressing hypertension in SSA, an *important limitation* of existing hypertension research is that the findings are largely based on cross-sectional data. Specifically, cross-sectional data are not informative about the incidence of hypertension as individuals age, and they cannot differentiate persistent hypertension from temporary episodes of elevated blood pressure. For example, age-gradients of hypertension

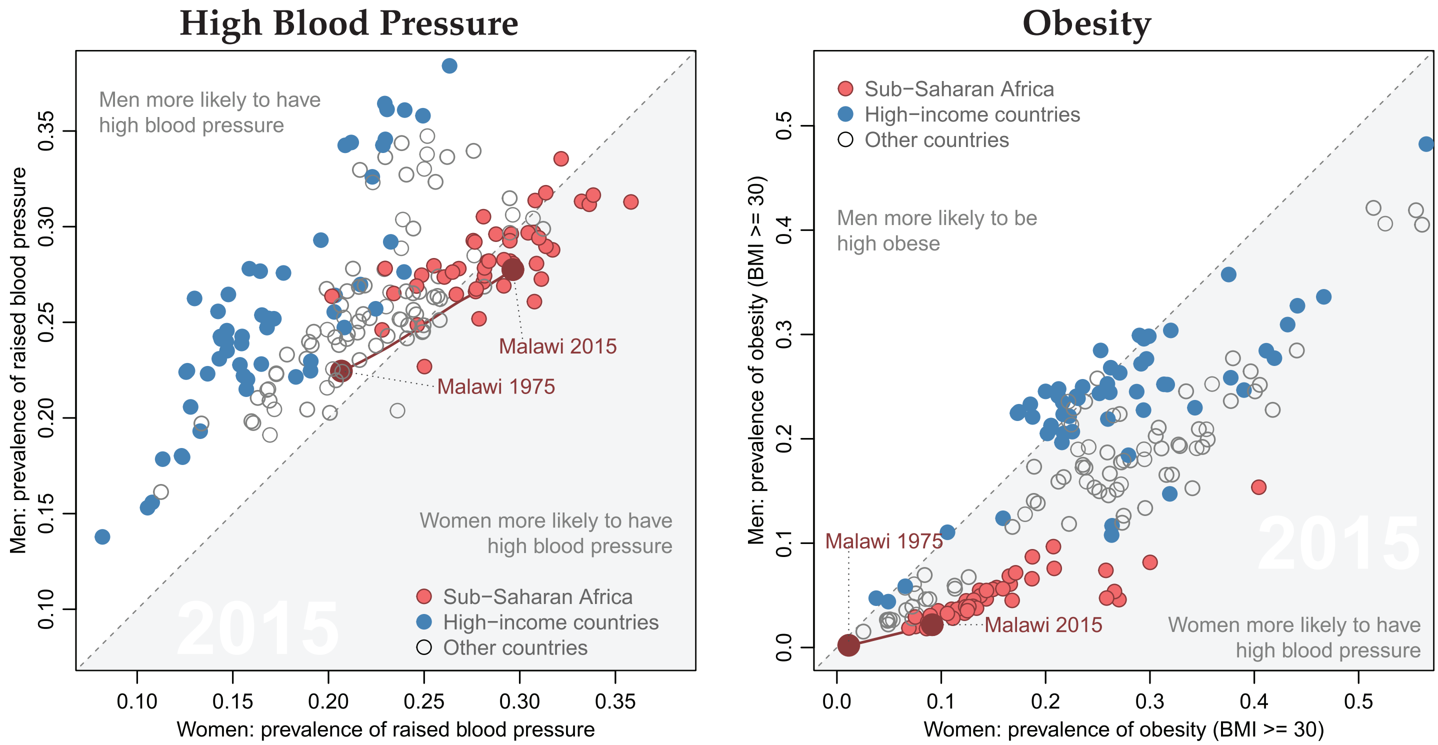

Fig 1. Prevalence of high blood pressure and obesity by sex across different regions in the world (2015, in %, age-standardized). *Source*: Own calculations based on NCD-RisC [1]. High blood pressure is defined by NCD-RisC as systolic BP $\geq$ 140 mmHg or diastolic BP $\geq$ 90 mmHg. Obesity is defined as BMI $\geq$ 30. Prevalence of high blood pressure and obesity are age-standardized so that they are better comparable across countries.

https://doi.org/10.1371/journal.pgph.0000600.g001

observed in cross-sectional data can arise from cohort differences, and such age gradients thus may not reflect changes in hypertension prevalence as individuals become older. Hence, if age-based increases in hypertension are used to create thresholds for targeting care efforts, cross-sectional data may misguide clinical decisions. Cross-sectional studies are also limited in their ability to identify the extent to which enhancing awareness and knowledge about blood pressure will improve blood pressure levels over time [12, 13]. Ultimately, only analyses of longitudinal data can provide a better understanding of hypertension as a dynamic process that is interrelated with individual aging and socioeconomic changes. Yet, very few such studies have been conducted in SSA, or more broadly in low-income countries (LICs).

The purpose of this paper is to provide among the first *longitudinal* analyses of widespread hypertension among aging global poor using *panel* data from a low-income country. The individuals that we study had lifecourse experiences that are typical for the aging global poor: They lived most of their lives with per capita incomes of less than $1/day [14]. Fertility was high in these cohorts [15], the status of women was generally low, and income generation through subsistence agriculture and small scale agriculture entailed relatively high levels of physical activity [16]. Older study participants were born when under-5 mortality was almost one out of three [5], and period life expectancies at birth were around 35 years in the 1950s, and remained below 46 years until the early 2000s [5]. All members of our study population have survived sustained poverty, frequent undernutrition, and epidemics. Health at older ages is often poor, and individuals experience accelerated aging with an early onset of morbidity across multiple domains [8, 17–19].

Due to the lack of longitudinal studies of hypertension in such low income populations, large gaps remain the literature about some basic aspects of hypertension risk during the lifecourse. Key research questions guiding our analyses for instance include: In this LIC population, do blood pressure and the prevalence of hypertension increases as individuals get older over time? What are socioeconomic predictors of the high blood pressure, or the onset of hypertension? Do patterns of hypertension and their predictors differ between men and women? What are possibly life-course consequences in terms of mortality or physical/mental health of hypertension? Do individuals in our LIC study population have knowledge about the health risk associated with hypertension? Finally, what can analyses of the cascades of care tell us about the diagnosis and treatment (or lack thereof) for individuals affected by hypertension?

While our paper does not directly test hypotheses related to these research questions, our results are useful for targeting hypertension prevention and care efforts to the populations with the most need and towards risk factors with the greatest potential benefit. For example, our results inform decisions about whether hypertension care should be targeted by age and sex, and they help clarify the degree to which intervention efforts should focus on addressing behavioral risk factors or improved medical treatment of hypertension.

Methods

Data

Data for this study come from the Mature Adults Cohort of the Malawi Longitudinal Study of Families and Health (MLSFH-MAC), which to our best knowledge is currently the only population-based cohort study of adults in a SSA LICs that collects *longitudinal information on blood pressure* along with data on hypertension prevalence, incidence, treatment, awareness and knowledge [16, 20]. The MLSFH is based in Malawi, one of the poorest countries in the world that is ranked 174 out of 189 countries in terms of the Human Development Index in 2019 (HDI_{2019} = .485) [21] and has a per-capita GDP equal to about 5% of the global average [22]. In rural areas, where our study is based and most Malawians (85%) live, about 60% of the

rural population was considered poor in 2016/17, thus having a total consumption that does *not* provide 2,400 calories per day per person [23].

The MLSFH-MAC study population consists of adults aged 45+ years living in rural areas in three districts in Malawi (Mchinji, Balaka, Rumphi). Baseline enrollment in 2012 was 1,266 individuals, and the cohort was followed up in 2013, 2017, and 2018, when each time additional eligible MLSFH respondents reaching age 45 years were enrolled in MLSFH-MAC. An MLSFH-MAC Cohort Profile provides detailed information about sampling procedures, study instruments, attrition/follow-up rates [16], and summary statistics for the study population are provided in S1 and S2 Tables. Life expectancy at birth is currently ≈63 years, and life expectancy at age 45 is currently ≈28 years [5]. Healthy life expectancy at age 45 is estimated around 22 years [8], and older adults can expect to live a large proportion of their later years subject to physical limitations on their activities [24].

We focus on MLSFH-MAC respondents with longitudinal measurements of blood pressure in 2013 (baseline) and the 2017 follow-up. The mean age is about 60 years in 2017, with 37% of respondents being age 45–54 years and 14% age 75+ years. 60% of the study population is female, and one quarter is Muslim. The study population is characterized by low levels of formal schooling (30% of respondents have no formal education, and 64% have only primary education). 58% of female and 94% of male respondents were married in 2017, and about half of the respondents resided in a house with a metal/tiled roof. The HIV prevalence for the MLSFH-MAC cohort was 8% in 2017, with HIV+ individuals concentrated at ages 45–49 years. The majority of respondents (66%) have body mass index (BMI) that is within the normal range ($18 \leq \text{BMI} \leq 25$) or are underweight (17% have BMI below 18). While only a small fraction of the study population is overweight (12%) or obese (4.7%), women are more likely to fall into these high risk BMI categories.

Measurement of blood pressure in MLSFH-MAC

Blood pressure (BP) in MLSFH-MAC was measured in 2013 and 2017 following the protocol developed by the Health and Retirement Study (HRS) [25]. Three BP measurements on the left arm were taken about 1 minute apart in a sitting position in a chair, using an upper arm automated BP monitor (Omron HEM-780N). BP data is available for 1,229 respondents aged 45+ in 2013 and 1,516 in 2017. Longitudinal BP measurements are available for 1,065 MLSFH-MAC participants. Respondents who measured BP above 160 mmHg systolic and/or 110 mmHg diastolic were given a referral for follow up with a doctor or medical professional, but were not provided any other information about blood pressure. Virtually all respondents (e.g., 99% of participants in 2017) agreed to the BP measurement. Prior to the BP measurement, respondents were asked if they have been diagnosed with hypertension by a doctor or medical personnel in the last two years before the survey, and if they are currently taking medication for reducing BP. Respondents' knowledge about the risks associated with high BP was elicited by asking why it is important for individuals to know their blood pressure, and the importance that respondents attributed to knowing their BP was assessed through a question asking how far (long) they would be willing to walk to a clinic to obtain a (free) BP measurement. Additionally, the 2017 survey included questions on knowledge about BP and associated risk factors, and a set of questions focused on the ability of respondents to recognize symptoms associated with high BP.

Approach

Individuals are defined as pre-hypertensive if $120 \leq BP_{sys} < 140$ or $80 \leq BP_{dia} < 90$, as hypertensive stage 1 if $140 \leq BP_{sys} < 160$ or $90 \leq BP_{dia} < 100$ and hypertensive stage 2 if $BP_{sys} \geq 160$

or $BP_{dia} \geq 100$, where BP_{sys} denotes systolic and BP_{dia} diastolic blood pressure. Onset of hypertension is defined as having normal blood pressure or pre-hypertension at baseline (2013), and having Stage 1 or 2 hypertension at follow-up in 2017. In 85% of cases, systolic BP determines the hypertension classification. Persistent hypertension is defined as being Stage 1 or Stage 2 hypertensive at both baseline (2013) and follow-up (2017). Persistent Stage 2 hypertension is defined as being Stage 2 hypertensive in both 2013 and 2017.

The "*cascade of care*" concept [26, 27] is used to measure linkage to care, health seeking behavior, and effective treatment for individuals with hypertension. The cascade represents the proportion of individuals who reach each separate stage of care, conditional on being included in the previous stage, and it is useful to identify the stages of the disease care cascade where the management of a disease fails. For hypertension care, the following cascade stages are defined: (1) being hypertensive (47% and 46% of all respondents in 2013 and 2017 respectively), either based on measured BP (90% and 84% of hypertensive cases in 2013 and 2017 respectively) and/or a reported diagnosis (22% or 39% of hypertensive cases in 2013 and 2017 respectively); (2) diagnosis of hypertension by a doctor or medical personnel within 2 years prior to the survey; (3) treatment of high BP with medication, and (4) treatment for high BP and controlled BP (= measured BP does not indicate hypertension at time of survey).

We employ standard regression analysis and focus on the results from the *longitudinal* analyses of hypertension during 2013–17, which are enabled by the longitudinal cohort information available in MLSFH-MAC. Related cross-sectional or complementary results are reported in the Supplemental Materials.

Ethics approval

This research based on the MLSFH-MAC was approved by the IRB at the University of Protocol #815016) and the College of Medicine (now Kamuzu University of Health Sciences) in Malawi (COMREC Protocol #P.01/12/1165).

Results

Prevalence of hypertension, hypertension incidence and persistent hypertension

At baseline in 2013, 42.5% of mature adults were hypertensive (23.5% Stage 1, 19% Stage 2; (Table 1, Column 1). In multivariate analyses, prevalence of hypertension is higher at older ages, is similar between women and men, varies across MLSFH study regions and is *not* associated with having obtained formal schooling (S3 Table).

Table 1. Prevalence of hypertension in 2013, and onset and persistence of hypertension during 2013–17 among MLSFH mature adults.

	2013 Prevalence of Hypertension		Change in Hypertension 2013–17 (Longitudinal Sample)		
	Baseline Sample[a] (1)	Longitud. Sample[b] (2)	Onset Hypertension (3)	Persistent Hypertension (4)	Persistent Stage 2 Hypertens. (5)
Normal blood pressure	25.3%	26.0%	10.5%	–	–
Pre-hypertension	32.1%	33.0%	28.5%	–	–
Stage 1 hypertension	23.5%	23.2%	–	59.1%	–
State 2 Hypertension	19.0%	17.8%	–	83.1%	58.4%
Obs (*N*)	1,229	1,065			

Notes:
[(a)] 2013 Baseline Sample = all MLSFH-MAC respondents with a 2013 blood pressure measurement;
[(b)] Longitudinal sample = all MLSFH-MAC respondents with a 2013 *and* 2017 blood pressure measurement

https://doi.org/10.1371/journal.pgph.0000600.t001

Hypertension prevalence is similar between the longitudinal sample (Table 1, Column 2), which is used for our cohort analyses of changes in hypertension 2013–17, and the cross-sectional sample at baseline in 2013 (Column 1), except for a lower prevalence of Stage 1 and Stage 2 hypertension that is due to selective survival (hypertension predicts mortality, but not other attrition, see below).

Cohort analyses (Table 1, Columns 3–5) show that 10.5% respondents with normal BP at baseline were hypertensive (Stage 1 or 2) by 2017; this proportion increases to 28.5% among those who were pre-hypertensive at baseline. 59% and 83% of respondents with Stage 1 or Stage 2 hypertension at baseline respectively were still hypertensive (Stage 1 or 2) by 2017. 58% of those with Stage 2 hypertension were still Stage 2 hypertensive by 2017. These patterns of prevalence, onset and persistence of hypertension among adults aged 45+ are very similar for men and women, and with one exception (onset of hypertension among respondents classified as pre-hypertensive at baseline), not statistically different (S4 Table).

Several factors are associated with the onset or persistence of hypertension among adults aged 45+ during 2013–17 (Table 2). There is a weak indication that women are more likely to have an onset of hypertension, but otherwise no clear gender difference in the persistence of hypertension over time. Being overweight or obese increases the odds of hypertension onset and the persistence of hypertension, and poor physical health (low SF12 physical score) and nutritional stress (no consumption of fish or meat in last seven days) predict the onset of hypertension. The same factors also predict changes in systolic BP among respondents not hypertensive at baseline, and in some cases also for the overall study population or other subgroups. Being HIV-positive or Muslim protects against increases in systolic BP. Being married is associated with increases in blood pressure and the persistence of hypertension, contrary to common findings that marriage often found to be protective for cardiovascular health [28]. Notably wealth (various indicators), schooling and grip strength are not predictors of the onset/persistence of hypertension or changes in blood pressure (S5 Table).

Blood pressure and aging

Table 3 shows only modest *within-person* increases in systolic blood pressure during 2013–17: For women aged 45–54 years there is an annual increase in systolic BP of.94 base points *per year of age* during 2013–17 (Column 1). This increase is similar to the age gradient in Western, but more than that in some other low-income populations [29]. There is no systematic age-related increase for men, or women aged 55+ (Columns 2–4). Diastolic blood pressure declines on average as respondents age. Identical conclusions are obtained comparing the adjacent box plots in Fig 2 that show 2013 and 2017 BP among longitudinally followed individuals: there is only minimal change in the distribution of systolic BP as cohort members aged during 2013–17, while diastolic BP declined as the cohort got older.

In combination, Table 3 and Fig 2 indicate that there is *no* increase in average hypertension risk in the MLSFH-MAC cohort as cohort members aged four years during 2013–17. While hypertension is highly prevalent and often persistent among MLSFH mature adults (Table 1), overall hypertension was *not* worsening in the MLSFH-MAC cohort: The distribution of hypertension categories in 2017 is indistinguishable from that in 2013 ($p(\chi^2) = .94$) for cohort members who survived and had BP measured at both waves (S6 Table).

Contrasting cohort and cross-sectional patterns in hypertension

In contrast to the relatively "*flat*" hypertension profile as the cohort got older during 2013–17, there is a strong *cross-sectional* increase in systolic BP and hypertension with age (Fig 2). For instance, 54.3% adults aged 45–54 had systolic BP of 120 or higher in 2013, and only 22% had

Table 2. Predictors of changes in systolic blood pressure, onset and persistence of hypertension.

	Among 2013 respondents who are						All respondents Combined	
	Not hypertensive		Hypertensive Stage 1 or 2		Hypertensive Stage 2			
	Onset of hypertension 2013–17 (Odds Ratio)	Change in systolic BP 2013–17 (OLS Coef)	Persistence of hypertension 2013–17 (Odds Ratio)	Change in systolic BP 2013–17 (OLS Coef)	Persistence of Stage 2 hypertension 2013–17 (Odds Ratio)	Change in systolic BP 2013–17 (OLS Coef)	Change in systolic BP 2013–17 (OLS Coef)	Change in diastolic BP 2013–17 (OLS Coef)
**Gender** (Ref.: Male)								
Female	1.45^{+}	0.99	1.28	1.43	1.57	5.31^{+}	1.23	1.24*
	[0.93,2.27]	[-1.44,3.42]	[0.86,1.91]	[-2.19,5.06]	[0.86,2.85]	[-0.27,10.9]	[-0.83,3.28]	[0.20,2.29]
**Body mass index (BMI), Categorical** (Ref.: Normal weight or underweight)								
Overweight/ obese	2.49**	4.72**	1.82^{+}	6.61**	2.88**	10.0*	4.55**	0.55
	[1.55,4.01]	[1.31,8.14]	[0.96,3.45]	[1.99,11.2]	[1.42,5.85]	[2.28,17.8]	[1.40,7.71]	[-1.06,2.16]
**Marital status** (Ref.: Not currently married)								
Currently married	1.19	1.49	1.88*	6.38*	2.86*	6.55	3.74*	1.45^{+}
	[0.70,2.02]	[-1.68,4.66]	[1.06,3.33]	[1.16,11.6]	[1.15,7.08]	[-1.64,14.7]	[0.70,6.78]	[-0.031,2.92]
**HIV status** (Ref.: HIV-negative)								
HIV-positive	0.24^{+}	-5.62**	0.63	-4.04	0.2	4.54	-4.61*	-3.79**
	[0.054,1.03]	[-9.65,-1.58]	[0.26,1.56]	[-12.4,4.36]	[0.019,2.11]	[-6.85,15.9]	[-8.82,-0.39]	[-6.43,-1.16]
**Religion** (Ref.: Christian)								
Muslim	0.70	-5.39**	1.02	-5.59*	0.67	-8.34	-5.57**	-1.25
	[0.31,1.59]	[-9.09,-1.69]	[0.47,2.23]	[-11.1,-0.065]	[0.29,1.56]	[-18.8,2.14]	[-8.65,-2.50]	[-3.53,1.02]
Other/none	0.73	1.30	0.58	-5.50	0.62	-4.50	-2.07	-0.41
	[0.25,2.10]	[-5.13,7.72]	[0.24,1.39]	[-15.3,4.33]	[0.18,2.19]	[-21.5,12.5]	[-6.74,2.59]	[-2.95,2.14]
**Low protein consumption** (Ref.: Ate fish or meat at least once in last 7 days)								
Ate no fish or meat in last 7 days	2.13**	5.56**	1.01	0.055	0.55	1.22	3.02^{+}	2.46**
	[1.36,3.34]	[2.42,8.70]	[0.52,1.99]	[-5.51,5.62]	[0.24,1.28]	[-7.05,9.49]	[-0.29,6.33]	[0.72,4.20]
**SF12 Physical health score** (z-score)								
SF12 Physical health z-score	0.77*	-2.34**	1.31*	0.34	1.15	-0.47	-1.48^{+}	-0.65
	[0.62,0.95]	[-4.00,-0.67]	[1.02,1.68]	[-2.45,3.14]	[0.79,1.68]	[-5.55,4.60]	[-3.03,0.074]	[-1.51,0.21]
Observations	≈625	≈620	≈435	≈410	≈190	≈175	≈1030	≈1030

Notes: Tables shows the results of OLS regressions (for change in blood pressure) or logistic regressions (for onset or persistence of hypertension) on the on the predictor variables, controlling additionally for age, gender and region. level. Analyses include all MLSFH-MAC respondents with both 2013 and 2017 BP measurement. Within-person change in BP is calculated as the difference of 2017 BP minus 2013 BP. Outliers in BP change are eliminated as they may reflect measurement errors (outliers are defined as being in the 1st or 99th percentile of the 2013–17 change in BP, *p*-values (robust std. errors): $p < 0.10$,

* $p < 0.05$,

** $p < 0.01$.

https://doi.org/10.1371/journal.pgph.0000600.t002

systolic BP of 140 or higher. At ages 75+, the respective factions are 78.1% ($p < .01$) and 52.5% ($p < .01$). The cross-sectional age gradient for systolic BP is.73 (CI: .95–.87) per year of age in 2013, and.077 (CI: .0062–.13) for diastolic BP (S3 Table). There is also a corresponding cross-sectional increase in hypertension with age: 30% of adults aged 45–54 were hypertensive (Stage 1 or 2) in 2013, increasing to 54% ($p < .01$) at ages 75+. A 10 year higher age increases the odds of being hypertensive in the cross-section by about 42% (CI: 32–54%), with similar increases also pertaining to of stage 2 hypertension (S3 Table). This cross-sectional patterns remains stable in 2017 (S3 Table).

The strong cross-sectional age gradient in BP is in contrast to the flat BP profile documented in our earlier longitudinal analyses (Table 3). Specifically, this divergence of the BP

Table 3. Average annual within-person change in blood pressure during 2013–17, by 2017 age group.

Age (2013):	45–54 (1)	55–64 (2)	65–74 (3)	75+ (4)	Pooled 45+ (5)
**Average annual within-person change in systolic BP:**					
Women	0.94**	0.14	0.41	-0.80	0.38+
	[0.45,1.44]	[-0.53,0.81]	[-0.77,1.59]	[-0.77,1.59]	[-0.033,0.79]
Men	0.03	0.27	-0.05	-0.22	0.07
	[-0.68,0.75]	[-0.50,1.04]	[-1.16,1.05]	[-1.60,1.15]	[-0.77,1.59]
**Average annual within-person change in diastolic BP:**					
Women	-0.14	-0.67**	-0.41	-0.63*	-0.42**
	[-0.43,0.14]	[-1.04,-0.30]	[-0.95,0.12]	[-1.24,-0.019]	[-0.62,-0.21]
Men	-0.61**	-0.92**	-0.76**	-0.53	-0.74**
	[-1.03,-0.20]	[-1.36,-0.48]	[-1.24,-0.28]	[-1.18,0.12]	[-0.97,-0.51]

p-values:

+ $p < 0.10$,

* $p < 0.05$,

** $p < 0.01$.

95% confidence intervals are estimated based on robust standard errors clustered at the village level. Analyses include all MLSFH-MAC respondents with both 2013 and 2017 BP measurement. Within-person change in BP is calculated as the difference of 2017 BP minus 2013 BP. Outliers in BP change are eliminated as they may reflect measurement errors (outliers are defined as being in the 1st or 99th percentile of the 2013–17 change in BP.

https://doi.org/10.1371/journal.pgph.0000600.t003

age-gradient between cohort and cross-sectional analyses is suggestive of potential cohort influences on BP and hypertension, that is, the possibility that the higher BP and hypertension prevalence among older adults is due to higher levels of life-course adversity experience by older persons. Such cohort effects are plausible because worse early-life conditions among

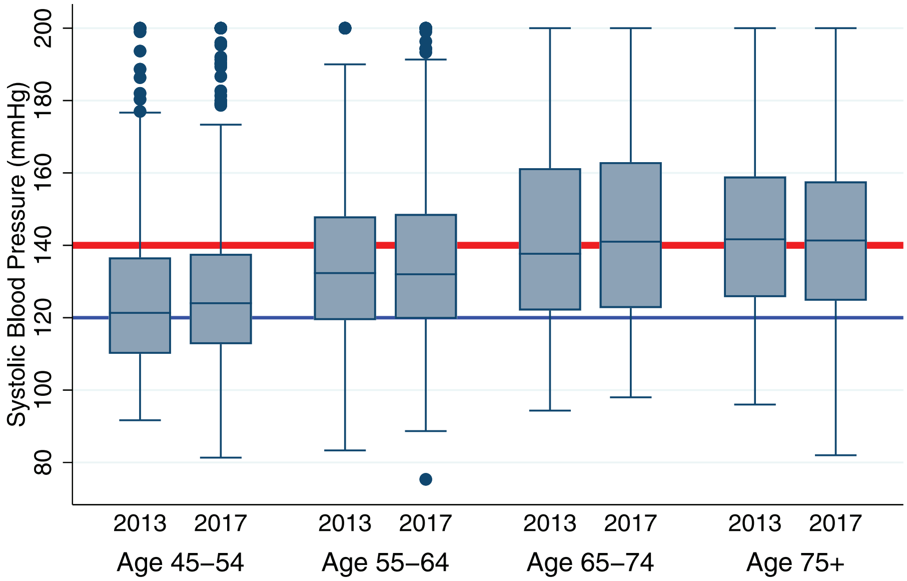

Fig 2. Systolic blood pressure (mmHg) among mature adults in 2013 and 2017, by age in 2013. *Notes*: Analyses include all MLSFH-MAC respondents with both 2013 and 2017 BP measurement. Age group is assigned based on age in 2013. Systolic BP is top-coded at 200 mmHg. The corresponding graph for diastolic BP is reported in S1 Fig. S2 and S3 Figs provide analyses separate for men and women.

https://doi.org/10.1371/journal.pgph.0000600.g002

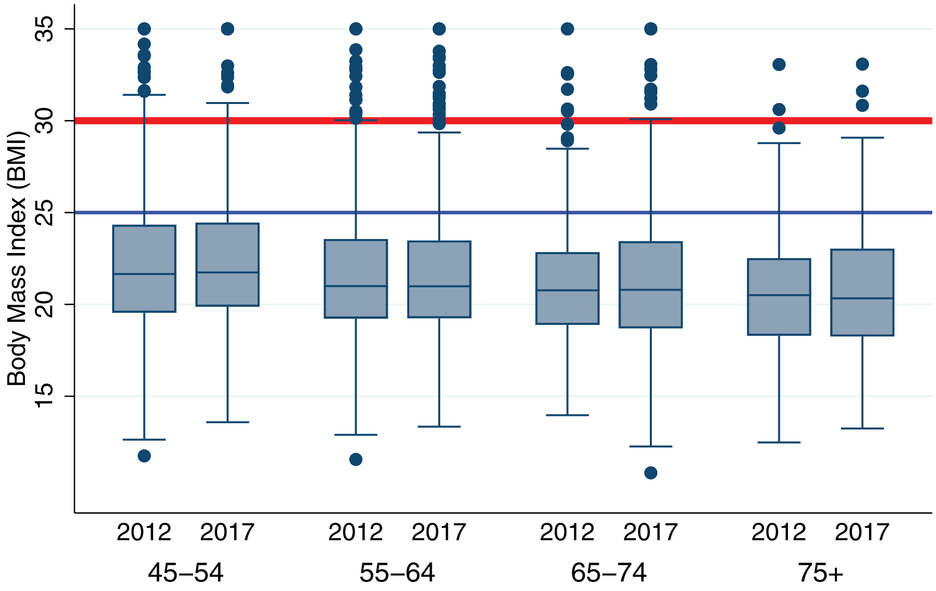

Fig 3. Body Mass Index (BMI) of MLSFH-MAC respondents aged 2012–13. *Notes*: Based on measured height and weight in 2012 and 2017. Red line denotes the cut-off for obesity (BMI≥30), and the blue line denotes the cut-off for overweight (25≤BMI≥30). Age group is assigned based on age in 2017. *Source*: Kohler et al. [16].

https://doi.org/10.1371/journal.pgph.0000600.g003

older cohorts has been linked to worse health outcomes at older ages (including worse cardiovascular health) [30, 31]. This aspect is especially important in countries having experienced socioeconomic development or improvements in health (and thus more pronounced cohort differences in early-life environments) during the 20th century [32, 33]. For example, height, a common indicator of early-life nutritional status that was measured in 2017, declines strongly in our study population with age (age-gradient = -.15cm, $p < .01$, with controls for region and gender).

Blood pressure, hypertension prevalence and body mass index

Our earlier analyses have shown that being overweight/obese or having high BMI is a risk factor factor for the onset and persistence of hypertension during 2013–17 (Table 2), as well as for having elevated blood pressure or being hypertensive (S2 Table). Yet, the overall high prevalence of hypertension among MLSFH Mature Adults contrasts sharply with an overall low prevalence of adiposity (Fig 3). Only a small fraction of respondents are overweight (12% of the total sample in 2017), and an even smaller fraction (4.7%) is obese. Above age 75, where the hypertension prevalence peaks, more than 25% of the respondents are underweight, and only few are obese (2%). There is neither a cross-sectional increase of BMI with age, nor a substantive within-person change in BMI during 2012–17. Age-related increases in BMI are *not* a factor driving the higher prevalence of hypertension at older ages, further confirming that other factors—such as worse early-life conditions or higher levels of life-course adversity—are contributing to the higher levels of hypertension among older persons.

Consequences of being hypertensive

Hypertension predicts mortality in the MLSFH-MAC cohort, with hypertensive individuals facing about 60% higher odds of dying during 2013–18 (Table 4, Column 1). Importantly for our cohort analyses, hypertension does *not* predict survey attrition for reasons other than

Table 4. Consequences of baseline hypertension in MLSFH-MAC cohort 2013–17.

Hypertension in 2013 (baseline)	**Mortality 2013–18** (Odds Ratios) (1)	**Non-mortality attrition** (Odds Ratios) (2)	**2013–17 Change in**: **Subjective health** (OLS Coef.) (3)	**SF12 Physical health score** (OLS Coef.) (4)	**PHQ9 Depression score** (OLS Coef.) (5)
Stage 1 Hypertension	1.24	1.28	0.013	-0.56	0.18
	[0.78,1.97]	[0.73,2.23]	[-0.17,0.20]	[-1.94,0.82]	[-0.52,0.88]
Stage 2 Hypertension	1.89**	1.03	-0.22*	-2.51**	1.04*
	[1.19,2.98]	[0.60,1.78]	[-0.40,-0.034]	[-4.20,-0.83]	[0.15,1.94]
Observations	1,225	1,227	1,108	1,104	1,108
	**2013–17 Change in**:				
	**GAD7 Anxiety score** (OLS Coef.) (6)	**Work efforts** (hours/wk) (OLS Coef.) (7)	**Grip Strength** (OLS Coef.) (8)	**Body Mass Index (BMI)** (OLS Coef.) (9)	**ICA Cognition score** (OLS Coef.) (10)
Stage 1 Hypertension	0.32	-1.57	-0.18	-0.025	0.13
	[-0.27,0.91]	[-4.25,1.11]	[-0.92,0.56]	[-0.40,0.35]	[-0.44,0.71]
Stage 2 Hypertension	0.96**	-6.37**	-0.13	0.11	-0.069
	[0.30,1.62]	[-9.19,-3.55]	[-0.94,0.68]	[-0.28,0.50]	[-0.81,0.68]
Observations	1,108	1,080	976	1,046	1,030

p-values:
+ $p < 0.10$,
* $p < 0.05$,
** $p < 0.01$.
95% confidence intervals are estimated based on robust standard errors clustered at the village level. Analyses additionally control for age and gender.

https://doi.org/10.1371/journal.pgph.0000600.t004

mortality (Column 2). Among survivors, hypertension at baseline is also associated with several adverse outcomes. For example, hypertension predicts declines in subjective health and SF12 physical health score relative to non-hypertensive cohort members during 2013–17, and it predicts (relative) increases in PHQ9 depression score and GAD7 anxiety scores (Columns 3–6). There is also evidence that hypertension reduces effort in common moderately-intensive work activities (e.g., time spent in animal care, firewood preparation, planting, etc.) (Table 4, Column 7). Baseline hypertension does not predict changes in physical health indicators such as grip strength or BMI during 2013–17, nor does it predict changes in cognitive scores (Columns 8–10).

Awareness of hypertension and knowledge of associated health risks

Respondents attribute significant value to knowing their BP, and around 60% report in 2013 a willingness to walk up to 1 hour to obtain a BP measurement, and 1/3 reports a willingness to walk more than 2 hours (Table 5). These reports are consistent with the almost universal consent to BP measurement as part of our study. This willingness does not increase among respondents who are hypertensive, but it is consistent with respondents associating BP with several dimensions of their overall health. However, not all of these associations are accurate. For instance, about 37% of respondents in 2013 (33% in 2017) think that knowing one's BP may indicate being HIV-positive (Table 5), an association that is not generally established [34, 35]. More than 70% of baseline respondent correctly state that BP knowledge can be informative about the with risk of stroke, heart disease, and diabetes. About 80% of respondents think BP is informative about their ability to work and being productive in daily life, in sharp contrast and the mostly asymptomatic nature of high BP (albeit there is some evidence in our study population that stage 2 hypertension leads to reductions in work effort; Table 4). About

Table 5. Awareness of hypertension and knowledge of the risks associated with high blood pressure in MLSFH-MAC cohort with 2013 and 2017 blood pressure measurement.

	Respondent is		
	**Not hypertensive (1)**	**Hypertensive (2)**	**Combined (3)**
If a government clinic offering blood pressure measurement were available nearby, would you get your blood pressure measured if you had to walk:			
1 hour	63%	59%	62%
2 hours	45%	45%	45%
More than 2 hours	36%	34%	35%
Why do you think that it might be important for individuals to know their blood pressure? It may tell them. . .			
**2013**:			
. . .if they have HIV	37%	35%	36%
. . .about their risk of stroke	76%	70%	73%
. . .something about how much they can work	78%	79%	79%
. . .if they have heart problems	87%	88%	87%
. . .if they have diabetes	70%	74%	72%
. . .if they have malaria	45%	47%	46%
**2017**:			
. . .if they have HIV	33%	29%	31%
. . .about their risk of stroke	64%	65%	64%
. . .something about how much they can work	71%	74%	72%
. . .if they have heart problems	73%	77%	75%
. . .if they have diabetes	46%	44%	45%
. . .if they have malaria	34%	33%	34%

https://doi.org/10.1371/journal.pgph.0000600.t005

46% of baseline respondents associate BP with malaria, an association that is empirically tenuous [36]. Notably, these assessments about the importance of knowing ones BP do not differ by respondent's own hypertension status: hypertensive individuals do *not* state more accurate reasons of why it is important to know BP, nor do they give more importance to BP knowledge. At follow-up in 2017, respondents report similar associations of blood pressure with other health outcomes as in 2013, albeit overall there is a declining perception that knowledge about BP can be indicative of other health issues.

Cascade of care for hypertension

There remain substantial gaps in the diagnosis and treatment of hypertension among adults aged 45+ in rural Malawi (Fig 4A). Among all respondents who were identified as hypertensive in 2013, only 22% have been diagnosed in the last 2 years by a doctor or medical professional as hypertensive (red bar). Among those who were diagnosed, less then 9% reported in 2013 to be taking treatment for hypertension (green bar); that is for about 61% of respondents their medical needs for disease treatment were not met in 2013. Among those who were on treatment, only 11% had BP levels "under control," i.e., within the normal range. The cascade for 2017 among cohort respondents shows an overall improved cascade (Fig 4B), but there remain substantial gaps in care: only 58% of respondents with measured high levels of BP were *not* diagnosed in the past as hypertensive, and 54% of the diagnosed do *not* report taking treatment. However, only 17% of hypertensive respondents have attained a measured BP in the normal range. In a related study we have has documented that prior testing—and specifically the receipt of referral letters to health care providers—helped reduce blood pressure, increase

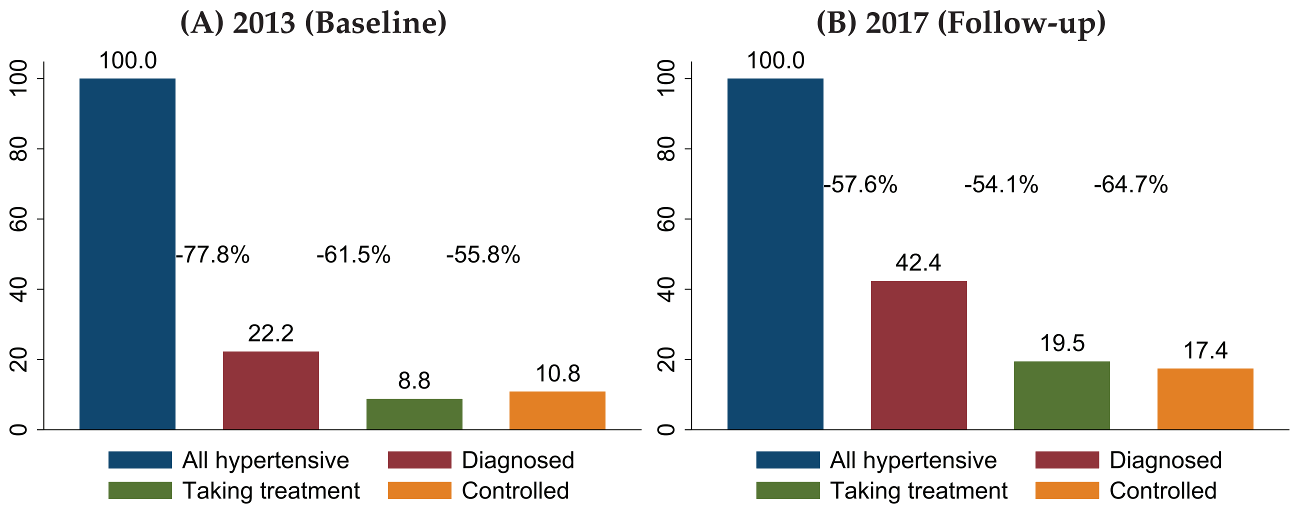

Fig 4. Cascade of care for hypertension in rural Malawi, cohort respondents age 45+ yrs. 2013–17. *Notes*: The cascade represents the proportion of individuals who reach each separate stage of care, conditional on being included in the previous stage. Analyses are based on respondents who participated in both the 2013 and 2017 MLSFH-MAC to ensure that variations in the study populations do *not* affect the findings; however, similar patterns are identified based on analyses of all 2013 or 2017 respondents.

https://doi.org/10.1371/journal.pgph.0000600.g004

diagnoses and update of medication [12] (similar findings have also been shown in South Africa [13]). The blood pressure screening that was conducted as part of the MLSFH-MAC may therefore have been instrumental in achieving improvements in case between 2013 and 2017 (Fig 4A+4B).

Summary and discussion

Cardiovascular diseases (CVDs) are rapidly becoming a major source of morbidity and mortality in many Sub-Saharan African (SSA) countries, while disease burdens attributable to communicable diseases are decreasing [8]. Addressing the implications of this shifting disease burden, which occurs together with a rapid aging of SSA populations, is critical for achieving the "grand convergence" in health that has been proposed as an achievable global health goal by 2035 [37]. Importantly, a renewed focus on NCDs—including hypertension as a top priority—will be critical as the Covid-19 pandemic has further revealed the incredible challenges of addressing a dual burden of communicable and non-communicable diseases in LICs [38], as well as the health and social vulnerabilities in LIC population that are caused by an often inadequately treated burden of noncommunicable diseases [39].

The rural subsistence agriculture population that is the focus of this study has low levels of modernization, very limited exposure to "classic" hypertension risk factors such as fast food consumption/unhealthy life styles, and is characterized by high levels of physical activity. Hypertension in such low-income populations has rarely been investigated using *longitudinal* data, and this is especially the case for older persons who are most at risk of cardiovascular diseases. Drawing on MLSFH-MAC *panel* data, this study is able to fill an important niche in the literature by highlighting *cohort patterns* of hypertension and highlighting the dynamics of onset and persistence of hypertension as individuals age during 2013–17.

Key findings of our analyses of blood pressure in this aging poor SSA population pertain to several domains of hypertension research: (1) ***Prevalence of hypertension and its predictors***: Hypertension is highly prevalent among individuals aged 45+, with more than 40% being hypertensive and more than 30% being pre-hypertensive. These high rates of hypertension

affect both men and women age 45+, in contrast to strong gender differences that have been documented in higher-income populations (Fig 1A). In our study population, the prevalence of hypertension is much higher than that of being overweight or obese, and while being overweight/obese is a predictor of hypertension, the higher prevalence of hypertension at older ages is not associated with higher levels of adiposity at older ages.

(2) ***Hypertension and aging—longitudinal changes in blood pressure***: (***a***) While there is a strong cross-sectional age gradients in blood pressure and hypertension, with older individuals having worse cardiovascular health, our longitudinal analyses using MLSFH-MAC data 2013–17 that on average a relatively flat profile of blood pressure or hypertension. As cohort members aged during 2012–17, there is no discernible increase in average blood pressure or the prevalence of hypertension: among longitudinally followed MLSFH-MAC respondents, the distribution of hypertension categories in 2017 is indistinguishable from that in 2013. The aging of the cohort during 2013–17 did therefore *not* increase average blood-pressure-related cardiovascular risk in this poor study population. (***b***) This divergence of cross-sectional and longitudinal age-gradient in BP and hypertension is likely due to cohort influences: earlier cohorts had worse early-life environments that, consistent with the Barker hypotheses [40], translated into worse cardiovascular health at older ages. (***c***) Despite the relatively stable distribution of blood pressure as the MLSFH-MAC cohort aged during 2021–17, blood-pressure-related cardiovascular risk is dynamic on the individual level. Among respondents with normal BP in 2013, about 10% experienced an onset of hypertension by 2017; among pre-hypertensive respondents in 2013, this rate almost triples and 28.5% experienced an onset of hypertension. (***d***) Stage 1 or 2 Hypertension, however, are often persistent: 59% of respondents with Stage 1 hypertension in 2013 were still hypertensive in 2017, and this is the case for 84% of respondents with Stage 2 hypertension at baseline in 2013. (***e***) The overall stable hypertension profile in the MLSFH-MAC cohort during 2013–17 arises because the onset of hypertension is compensated by who experience a decline in blood pressure or a transition from being hypertensive to normal blood pressure or pre-hypertension.

(3) ***Predictors of onset of hypertension over time***: (***a***) Being overweight/obese is a strong predictor of the onset and persistence of hypertension during 2012–17, and poor physical health (low S12 physical score) and facing nutritional stress (not having eaten fish or meat in last 7 days at baseline) predicts the onset of hypertension. Being HIV-positive or Muslim implies lower odds of experiencing increases in systolic BP. Interestingly, marriage may not be protective for cardiovascular health, in contrast to a common finding in other populations [28]. Being currently married in our study population is associated with increasing blood pressure over time and the persistence of hypertension. (***b***) Nevertheless, there are few systematic *socioeconomic* predictors of the onset or persistence of hypertension in this study population aged 45+ during 2013–17; notably neither schooling, wealth, nor grip strength (all measured in 2013) predict the onset/persistence of hypertension during 2013–17 or *changes* in systolic/diastolic blood pressure (albeit these factors predict cross-sectional *level* differences in BP or hypertension prevalence in the expected direction).

(4) ***Life-course consequences of hypertension***: Importantly, hypertension is has measurable consequences among adults aged 45+ in Malawi. Hypertension for example predicts mortality in the MLSFH-MAC population during 2013–17, with hypertensive individuals facing about 60% higher odds of dying. Yet, hypertension is not associated with non-mortality attrition in the MLSFH-MAC. Hypertension at baseline also associated with *declines* in subjective health, SF12 physical health scores and work efforts during 2013–17 and it is associated with *increases* in depression and anxiety. These findings thus reaffirm that hypertension is an important public health concern among adults aged 45+ in rural Malawi that is associated with more rapid declines in health as individuals get older.

(5) ***Cascade of hypertension care***: While diagnoses and treatment of hypertension are improving, our analyses of the cascade of care document that there remain substantial gaps in the diagnosis and treatment of hypertension among adults aged 45+ in rural Malawi. In 2013, for example, only 22% have been diagnosed in the last two years by a doctor or medical professional as hypertensive, and among those who were diagnosed, less then 9% reported in 2013 to be taking treatment for hypertension. Yet, this cascade of care substantially improved by 2017. 43% of hypertensive individuals were diagnosed, and 46% of the diagnosed were taking treatments. Yet, despite these improvements in the cascade of care, only 17% of hypertensive respondents have attained a measured BP in the normal range. These gaps in the cascade of care are disconcerting as high blood pressure is one of the main modifiable causes of cardiovascular disease risk with a large body of evidence showing that lowering blood pressure through low-cost and widely available medications significantly reduces CVD mortality [41]. The findings of our cascade-of-care analyses are also important as they help inform possible primary health care interventions that can increase management and treatment of hypertension and related chronic non-communicable diseases. Importantly, prior research [42, 43] indicates that the chronicity of these diseases necessitates a restructuring of healthcare to address the multidisciplinary, sustained care including psychosocial support and development of self-management skills, with primary healthcare providers offering promising opportunities for targeted interventions, including for instance by increasing contact of at-risk populations with the health care system (including through screening interventions [7, 12, 13]), improvements of awareness, equipment (e.g., blood pressure monitors) and access to medications among health care providers [44], updates/enhancements of the primary care guidelines to better reflect the needs of older persons [43] or other at-risk individuals (e.g., by integrating hypertension and HIV management [45]), improved linkage to care using cell phone and related technologies [46], and other improvements in the longitudinal control of hypertension [47, 48].

Overall, our findings in this paper indicate that hypertension and related high cardiovascular risks are widespread, persistent and often not diagnosed or treated in this rural sub-Saharan population aged 45+. The prevalence, onset and persistence of hypertension cuts across all subgroups in this population—including, importantly, both women and men. While age is an important predictor of hypertension risk, as has been documented in other LMIC contexts [7], hypertension is already widespread in in middle ages 45–55 years. Hence, hypertension among adults 45+ in Malawi seems to be more similar to a "generalized epidemic" than in high-income countries where cardiovascular risk has strong socioeconomic gradients and untreated hypertension particularly prevalent in vulnerable subsets of older persons [49].

Yet, our findings do not provide a bleak picture about hypertension and cardiovascular disease risk in poor aging populations in SSA or elsewhere. In contrast, a recent body of research has started to provide evidence that relatively inexpensive screenings and referrals for hypertension and are effective approach to reduce the gaps in the cascade of care for hypertension [12, 13]. Our findings highlight the urgency of building on this recent evidence and expand information about cardiovascular risk, screening for hypertension and available treatments for elevated blood pressure to the global poor. There are also primary healthcare intervention that can be scaled up at modest costs [42, 43]. Such screening efforts or primary healthcare enhancements are likely to have large returns in terms of improving population health among older persons [37].

Some limitations of our study are noteworthy. While the MLSFH-MAC is based on a large, population-based sample of adults 45+ years old living in a rural SSA LIC context, it is not a nationally representative sample. This concern is somewhat ameliorated by the fact that the MLSFH-MAC cohort characteristics closely match that of cross-sectional nationally-representative samples [16] and the fact that roughly 85% of the Malawian population resides in rural

areas [50]. Hence, our findings can likely be generalized to other rural areas in Malawi and similar low-income populations in southeastern SSA. The age range covered in our study (age 45+ years) is also comparable to other aging studies in SSA LICs [51], and given life expectancy trends in Malawi and SSA more generally, the study represents adequately the experience of the older population in the region. As in any longitudinal study, attrition is a concern for the MLSFH. However, non-mortality-related attrition has been very low in the MLSFH-MAC as a result of migration tracking and related fieldwork procedures; during 2012–18 the MLSFH-MAC retained 97% of surviving respondents [16]; importantly, non-mortality-related attrition 2013–17 is *not* associated with hypertension. Detailed analyses of attrition as part of the MLSFH cohort profiles [16, 20] have also shown that, even though respondent characteristics often differ significantly between those who were lost to follow-up and those who were re-interviewed, the coefficient estimates for standard family background variables in regressions and probit equations for many health outcomes are not significantly affected by attrition.

Population-based blood pressure measurement in rural older and poor populations in challenging, and measurements of blood pressure on a single session—as is conducted in most population-based health surveys, including the MLSFH—does not provide a clinical diagnoses of hypertension. Yet the MLSFH-MAC adopted the protocol for Health and Retirement Study [25], and through extensive training and carefully-developed fieldwork logistics, the MLSFH-MAC not only achieved very high rates of consent to blood pressure measurements, and data quality of the blood pressure measures is generally high [16]. However, the MLSFH-MAC data lack details about how individuals are affected by hypertension, and among those who are treatment, what specific type of hypertension they receive and/or how frequently they are followed up by the health system.

Given the scarcity of longitudinal data on blood pressure and hypertension in SSA low-income populations, the limitations of this study are relatively minor. The MLSFH-MAC is one of the rare cohort studies in a SSA LICs that provides detailed longitudinal information about blood pressure, hypertension knowledge and treatment, and our analyses are among the first that document the *cohort patterns* of hypertension in an aging SSA LIC population.

Supporting information

S1 Fig. Diastolic blood pressure (mmHg) among mature adults in 2013 and 2017, by age in 2013.
(EPS)

S2 Fig. Systolic blood pressure (BP mmHg) among mature adults in 2013 and 2017, by gender and age in 2013. *Notes*: Analyses include all MLSFH-MAC respondents with both 2013 and 2017 BP measurement. Age group is assigned based on age in 2013. Systolic BP is top-coded at 200 mmHg.
(EPS)

S3 Fig. Diastolic blood pressure (BP mmHg) among mature adults in 2013 and 2017, by gender and age in 2013. *Notes*: Analyses include all MLSFH-MAC respondents with both 2013 and 2017 BP measurement. Age group is assigned based on age in 2013. Diastolic BP is top-coded at 110 mmHg.
(EPS)

S1 Table. Summary statistics for the MLSFH mature adult cohort in 2017 (respondents with BP measurement), and bivariate associations with systolic and diastolic blood pressure.
(EPS)

S2 Table. Health indicators for the MLSFH mature adult cohort in 2017 (respondents with BP measurement), and associations with systolic and diastolic blood pressure.
(EPS)

S3 Table. Cross-sectional association of blood pressure and hypertension with age, 2013 and 2017.
(EPS)

S4 Table. Prevalence of hypertension in 2013, by gender, and onset and persistence of hypertension during 2013–17, by gender.
(EPS)

S5 Table. Predictors of changes in systolic blood pressure, onset and persistence of hypertension (additional findings).
(EPS)

S6 Table. Prevalence of hypertension in 2013 and 2017 among MLSFH mature adults with blood pressure measurement in both years.
(EPS)

Author Contributions

Conceptualization: Iliana V. Kohler, Nikkil Sudharsanan, Hans-Peter Kohler.

Data curation: Iliana V. Kohler.

Formal analysis: Iliana V. Kohler, Nikkil Sudharsanan, Hans-Peter Kohler.

Funding acquisition: Iliana V. Kohler, Hans-Peter Kohler.

Investigation: Iliana V. Kohler, Hans-Peter Kohler.

Methodology: Iliana V. Kohler, Hans-Peter Kohler.

Project administration: Iliana V. Kohler, Hans-Peter Kohler.

Writing – original draft: Iliana V. Kohler, Hans-Peter Kohler.

Writing – review & editing: Nikkil Sudharsanan, Chiwoza Bandawe, Hans-Peter Kohler.

References

1. NCD Risk Factor Collaboration (NCD-RisC). Worldwide trends in blood pressure from 1975 to 2015: A pooled analysis of 1479 population-based measurement studies with 19.1 million participants. Lancet. 2017; 389(10064):37–55. https://doi.org/10.1016/S0140-6736(16)31919-5 PMID: 27863813
2. NCD Risk Factor Collaboration (NCD-RisC). Worldwide trends in hypertension prevalence and progress in treatment and control from 1990 to 2019: a pooled analysis of 1201 population-representative studies with 104 million participants. Lancet. 2021; 398(10304):957–980. https://doi.org/10.1016/S0140-6736(21)01330-1
3. Geldsetzer P, Manne-Goehler J, Marcus ME, Ebert C, Zhumadilov Z, Wesseh CS, et al. The state of hypertension care in 44 low-income and middle-income countries: a cross-sectional study of nationally representative individual-level data from 1.1 million adults. Lancet. 2019; 394(10199):652–662. https://doi.org/10.1016/S0140-6736(19)30955-9 PMID: 31327566
4. The Economist. Daily chart: The curious case of high blood pressure around the world. Economist. 2017. Available from: https://www.economist.com/graphic-detail/2017/01/13/the-curious-case-of-high-blood-pressure-around-the-world
5. United Nations Population Division. World Population Prospects, the 2019 Revision; 2019. Available from: https://population.un.org/wpp/.

6. Sudharsanan N, Geldsetzer P. Impact of Coming Demographic Changes on the Number of Adults in Need of Care for Hypertension in Brazil, China, India, Indonesia, Mexico, and South Africa. Hypertension. 2019; 73(4):770–776. https://doi.org/10.1161/HYPERTENSIONAHA.118.12337 PMID: 30739534

7. Kirschbaum TK, Theilmann M, Sudharsanan N, Manne-Goehler J, Lemp JM, De Neve JW, et al. Targeting Hypertension Screening in Low- and Middle-Income Countries: A Cross-Sectional Analysis of 1.2 Million Adults in 56 Countries. Journal of the American Heart Association. 2021; 10(13):e021063. https://doi.org/10.1161/JAHA.121.021063 PMID: 34212779

8. GBD 2019 Disease Collaboration. Global burden of 369 diseases and injuries in 204 countries and territories, 1990–2019: a systematic analysis for the Global Burden of Disease Study 2019. Lancet. 2020; 396(10258):1204–1222. https://doi.org/10.1016/S0140-6736(20)30925-9

9. Kämpfen F, Wijemunige N, Evangelista B. Aging, non-communicable diseases, and old-age disability in low- and middle-income countries: a challenge for global health. International Journal of Public Health. 2018; 63(9):1011–1012. https://doi.org/10.1007/s00038-018-1137-z PMID: 29951746

10. Greenberg H, Raymond SU, Leeder SR. The Prevention of Global Chronic Disease: Academic Public Health's New Frontier. American Journal of Public Health. 2011; 101(8):1386–1390. https://doi.org/10.2105/AJPH.2011.300147 PMID: 21680924

11. Herrick C, Reubi D. The future of the global noncommunicable disease agenda after Covid-19. Health & Place. 2021; 71:102672. https://doi.org/10.1016/j.healthplace.2021.102672 PMID: 34543839

12. Ciancio A, Kämpfen F, Kohler HP, Kohler IV. Health Screening for Emerging Disease Burdens Among the Global Poor: Evidence from Sub-Saharan Africa. Journal of Health Economics. 2021; 75. https://doi.org/10.1016/j.jhealeco.2020.102388 PMID: 33249266

13. Sudharsanan N, Chen S, Garber M, Bärnighausen T, Geldsetzer P. The Effect Of Home-Based Hypertension Screening On Blood Pressure Change Over Time In South Africa. Health Affairs. 2020; 39 (1):124–132. https://doi.org/10.1377/hlthaff.2019.00585 PMID: 31905068

14. Malawi National Statistical Office. Malawi Poverty Report 2018. Lilongwe, Malawi: Government of Malawi, National Statistical Office (NSO); 2018. Available from: http://www.nsomalawi.mw/images/stories/data_on_line/economics/poverty/Malawi%20Poverty%20Report%20-%202019%20.pdf.

15. Kohler IV, Kohler HP, Anglewicz P, Behrman JR. Intergenerational Transfers in the Era of HIV/AIDS: Evidence from Rural Malawi. Demographic Research. 2012; 27(27):775–834. https://doi.org/10.4054/demres.2012.27.27 PMID: 23606809

16. Kohler IV, Bandawe C, Ciancio A, Kämpfen F, Payne C, Mwera J, et al. Cohort Profile: The Mature Adults Cohort of the Malawi Longitudinal Study of Families and Health (MLSFH-MAC). BMJ Open. 2020; 10:e038232. https://doi.org/10.1136/bmjopen-2020-038232 PMID: 33067285

17. Koyanagi A, Lara E, Stubbs B, Carvalho AF, Oh H, Stickley A, et al. Chronic Physical Conditions, Multimorbidity, and Mild Cognitive Impairment in Low- and Middle-Income Countries. Journal of the American Geriatrics Society. 2018; 66(4):721–727. https://doi.org/10.1111/jgs.15288 PMID: 29427504

18. Bollyky TJ, Templin T, Cohen M, Dieleman JL. Lower-Income Countries That Face The Most Rapid Shift In Noncommunicable Disease Burden Are Also The Least Prepared. Health Affairs. 2017; 36 (11):1866–1875. https://doi.org/10.1377/hlthaff.2017.0708 PMID: 29137514

19. WHO. World Report on Ageing and Health. Geneva, Switzerland: World Health Organization (WHO); 2015. Available from: http://www.who.int/ageing/publications/world-report-2015/en/.

20. Kohler HP, Watkins SC, Behrman JR, Anglewicz P, Kohler IV, Thornton RL, et al. Cohort Profile: The Malawi Longitudinal Study of Families and Health (MLSFH). International Journal of Epidemiology. 2015; 44(2):394–404. https://doi.org/10.1093/ije/dyu049 PMID: 24639448

21. United Nations Development Programme. Human Development Report 2020: Briefing Notes for Malawi; 2020. Available from: http://hdr.undp.org/sites/all/themes/hdr_theme/country-notes/MWI.pdf.

22. World Bank. World Bank Open Data: GNI per capita, Atlas method (current US$); 2020. Available from: https://data.worldbank.org/indicator/NY.GNP.PCAP.CD?locations=MW-1W&view=chart.

23. Malawi National Statistical Office. Preliminary Report: 2018 Malawi Population and Housing Census: Be Counted. Leave No One Behind. Lilongwe, Malawi: Government of Malawi, National Statistical Office (NSO); 2018. Available from: http://www.nsomalawi.mw/images/stories/data_on_line/demography/census_2018/2018%20Population%20and%20Housing%20Census%20Preliminary%20Report.pdf

24. Payne C, Mkandawire J, Kohler HP. Disability Transitions and Health Expectancies Among Adults 45 Years and Older Mature in Malawi: A Cohort Modeling Approach. PLOS Medicine. 2013; 10(5): e1001435. https://doi.org/10.1371/journal.pmed.1001435 PMID: 23667343

25. Health and Retirement Study (HRS). HRS 2010 Study Materials (CD for training interviewers and data collectors); 2010.

26. Stokes A, Berry KM, Mchiza Z, Parker W, Labadarios D, Chola L, et al. Prevalence and unmet need for diabetes care across the care continuum in a national sample of South African adults: Evidence from the SANHANES-1, 2011-2012. PLOS ONE. 2017; 12(10):e0184264. https://doi.org/10.1371/journal.pone.0184264 PMID: 28968435

27. Ali MK, Bullard KM, Gregg EW, Del Rio C. A cascade of care for diabetes in the United States: Visualizing the gaps. Annals of Internal Medicine. 2014; 161(10):681–689. https://doi.org/10.7326/M14-0019 PMID: 25402511

28. Wong CW, Kwok CS, Narain A, Gulati M, Mihalidou AS, Wu P, et al. Marital status and risk of cardiovascular diseases: a systematic review and meta-analysis. Heart. 2018; 104(23):1937. https://doi.org/10.1136/heartjnl-2018-313005 PMID: 29921571

29. Gurven M, Blackwell AD, Rodriguez DE, Stieglitz J, Kaplan H. Does Blood Pressure Inevitably Rise With Age? Hypertension. 2012; 60(1):25–33. https://doi.org/10.1161/HYPERTENSIONAHA.111.189100 PMID: 22700319

30. Barker DJP. Fetal origins of coronary heart disease. British Medical Journal. 1995; 311(6998):171. https://doi.org/10.1136/bmj.311.6998.171 PMID: 7613432

31. Hoffman DJ, Reynolds RM, Hardy DB. Developmental origins of health and disease: current knowledge and potential mechanisms. Nutritrion Reviews. 2017; 75(12):951–970. https://doi.org/10.1093/nutrit/nux053 PMID: 29186623

32. Engle PL, Black MM, Behrman JR, de Mello MC, Gertler Kapiriri L, et al. Strategies to avoid the loss of developmental potential in more than 200 million children in the developing world. Lancet. 2007; 369 (9557):229–242. https://doi.org/10.1016/S0140-6736(07)60112-3 PMID: 17240290

33. Grantham-McGregor S, Cheung YB, Cueto S, Glewwe P, Richter L, Strupp B. Developmental potential in the first 5 years for children in developing countries. Lancet. 2007; 369(9555):60–70. https://doi.org/10.1016/S0140-6736(07)60032-4 PMID: 17208643

34. Jericó C, Knobel H, Montero M, Sorli ML, Guelar A, Gimeno JL, et al. Hypertension in HIV-Infected Patients: Prevalence and Related Factors. Amerian Journal of Hypertension. 2005; 18(11):1396–1401. https://doi.org/10.1016/j.amjhyper.2005.05.016 PMID: 16280270

35. Dillon DG, Gurdasani D, Riha J, Ekoru K, Asiki G, Mayanja BN, et al. Association of HIV and ART with cardiometabolic traits in sub-Saharan Africa: a systematic review and meta-analysis. International Journal of Epidemiology. 2014; 42(6):1754–1771. https://doi.org/10.1093/ije/dyt198

36. Eze IC, Bassa FK, Essé C, Koné S, Acka F, Laubhouet-Koffi V, et al. Epidemiological links between malaria parasitaemia and hypertension: findings from a population-based survey in rural Côte d'Ivoire. Journal of Hypertension. 2019; 37(7):1384–1392. https://doi.org/10.1097/HJH.0000000000002071 PMID: 30801386

37. Jamison DT, Summers LH, Alleyne G, Arrow KJ, Berkley S, Binagwaho A, et al. Global health 2035: A world converging within a generation. Lancet. 2013; 382(9908):1898–1955. https://doi.org/10.1016/S0140-6736(13)62105-4 PMID: 24309475

38. Collins T, Tello J, Van Hilten M, Mahy L, Banatvala N, Fones G, et al. Addressing the double burden of the COVID-19 and noncommunicable disease pandemics: a new global governance challenge. International Journal of Health Governance. 2021; 26(2):199–212. https://doi.org/10.1108/IJHG-09-2020-0100

39. WHO. The impact of the COVID-19 pandemic on noncommunicable disease resources and services: results of a rapid assessment. Geneva, Switzerland: World Health Organization WHO; 2020. Available from: https://www.who.int/teams/noncommunicable-diseases/covid-19.

40. Barker D, Eriksson J, Forsen T, Osmond C. Fetal origins of adult disease: strength of effects and biological basis. International Journal of Epidemiology. 2002; 31:1235–1239. https://doi.org/10.1093/ije/31.6.1235 PMID: 12540728

41. GBD 2017 Risk Factor Collaborators. Global, regional, and national comparative risk assessment of 84 behavioural, environmental and occupational, and metabolic risks or clusters of risks for 195 countries and territories, 1990–2017: a systematic analysis for the Global Burden of Disease Study 2017. Lancet. 2018; 392(10159):1923–1994.

42. Macinko J, Leventhal DGP, Lima-Costa MF. Primary Care and the Hypertension Care Continuum in Brazil. Journal of Ambulatory Care Management. 2018; 41(1):34–46. https://doi.org/10.1097/JAC.0000000000000222 PMID: 29176460

43. Lall D, Prabhakaran D. Organization of primary health care for diabetes and hypertension in high, low and middle income countries. Expert Review of Cardiovascular Therapy. 2014; 12(8):987–995. https://doi.org/10.1586/14779072.2014.928591 PMID: 24934722

44. Wood R, Van Der Merwe L, Viljoen V, Mash R. Quality of care for patients with non-communicable diseases in the Dedza district, Malawi. African Journal of Primary Health Care and Family Medicine. 2015; 7(1):1–8. https://doi.org/10.4102/phcfm.v7i1.838 PMID: 26245609

45. Patel P, Speight C, Maida A, Loustalot F, Giles D, Phiri S, et al. Integrating HIV and hypertension management in low-resource settings: Lessons from Malawi. PLOS Medicine. 2018; 15(3):e1002523. https://doi.org/10.1371/journal.pmed.1002523 PMID: 29513674

46. Piette JD, Datwani H, Gaudioso S, Foster SM, Westphal J, Perry W, et al. Hypertension Management Using Mobile Technology and Home Blood Pressure Monitoring: Results of a Randomized Trial in Two Low/Middle-Income Countries. Telemedicine and e-Health. 2012; 18(8):613–620. https://doi.org/10.1089/tmj.2011.0271 PMID: 23061642

47. Mauer N, Geldsetzer P, Manne-Goehler J, Davies J, Stokes AC, McConnell M, et al. Treatment discontinuation and loss of hypertension control: Longitudinal evidence from population-based cohorts in four middle-income countries; 2022.

48. Ali MK, Chwastiak L, Poongothai S, Emmert-Fees KMF, Patel SA, Anjana RM, et al. Effect of a collaborative care Model on Depressive Symptoms and glycated Hemoglobin, Blood Pressure, and Serum Cholesterol Among Patients With Depression and Diabetes in India: The INDEPENDENT Randomized Clinical Trial. JAMA. 2020; 324(7):651–662. https://doi.org/10.1001/jama.2020.11747 PMID: 32809002

49. Leng B, Jin Y, Li G, Chen L, Jin N. Socioeconomic status and hypertension: a meta-analysis. Journal of Hypertension. 2015; 33(2):221–229. https://doi.org/10.1097/HJH.0000000000000428 PMID: 25479029

50. Malawi National Statistical Office. 2018 Malawi Population and Housing Census: Main Report. Zomba, Malawi: Malawi National Statistical Office (Malawi NSO); 2019. Available from: http://www.nsomalawi.mw/images/stories/data_on_line/demography/census_2018/2018%20Malawi%20Population%20and%20Housing%20Census%20Main%20Report.pdf.

51. Brinkmann B, Payne CF, Kohler I, Harling G, Davies J, Witham M, et al. Depressive symptoms and cardiovascular disease: a population-based study of older adults in rural Burkina Faso. BMJ Open. 2020; 10(12):e038199. https://doi.org/10.1136/bmjopen-2020-038199 PMID: 33371016
